# Supplementary material for: Observation of suppressed viscosity in the normal state of 3He due to superfluid fluctuations
Source: Nat Commun. 2023 Sep 20;14:5834. doi: 10.1038/s41467-023-41422-3 (PMC10511454; doi:10.1038/s41467-023-41422-3)
Supplement: Supplementary file 1 — Supplementary Information [file 41467_2023_41422_MOESM1_ESM.pdf]

Supplementary Information: Observation of suppressed viscosity in the normal state of ^{3}He due to superfluid fluctuations

R.N. Baten[1], Y. Tian[1], E.N. Smith[1], E. Mueller[1], and J.M. Parpia*[1]

[1]*Department of Physics, Cornell University, Ithaca, NY, 14853, USA*

August 30, 2023

Supplementary Note 1: Calculation of $\tau_\eta T^2$ and other parameters

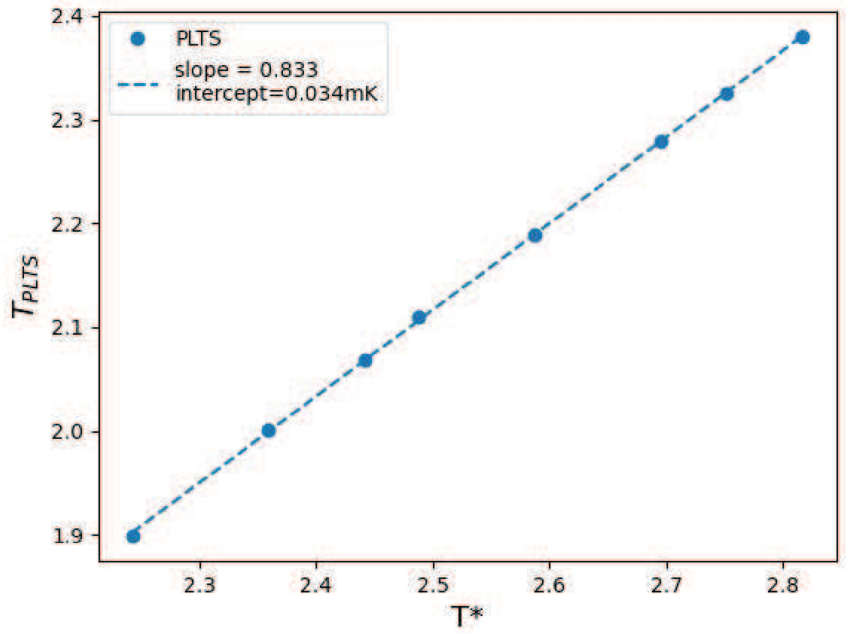

Supplementary Figure 1: **Conversion from T^* to T_{PLTS}.** Values of T_c^* [1,2] plotted against values of T_c on the $PLTS$ scale[3,4].

To arrive at an estimate for the magnitude of the fluctuation contribution to the Q, we need to determine various pressure dependent quantities for liquid ^{3}He. We start with the determination of $\tau_\eta T^2$, the quasiparticle scattering time associated with the viscosity. The Fermi liquid viscosity was studied by Parpia and co-workers[1,2]. The temperature scale used in that work needs to be converted to the PLTS scale[3]. We plot the values of T_c^* against the values of T_c in the PLTS scale[4] in Supplementary Figure 1. The conversion requires a linear scaling with a small offset, yielding $T_{PLTS} = 0.833T^* + 0.034$. The pressure dependent viscosity coefficients ηT^2 [poise-mK^2] listed in Ref[1] are then converted to their values with the PLTS scale.

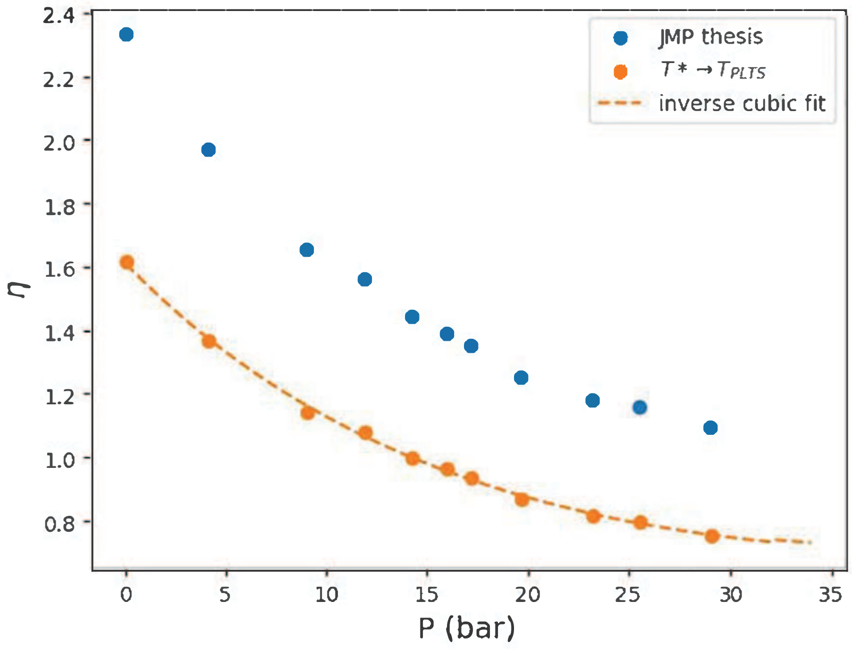

Supplementary Figure 2: ηT^2 **vs Pressure**. Values of the viscosity coefficient[1] before conversion (blue) and after conversion to the PLTS scale (gold). The dashed line is a cubic fit to $\eta(P)T^2$.

The viscosity coefficient, $\eta(P)T^2$ (η in poise, P in bar, T in mK following the PLTS scale), shown in Supplementary Figure 2 and listed in Supplementary Table 1, can be calculated from the relation,

$$(\eta(P)T^2)^{-1} = \sum_{i=0}^{3} A_i P^i, \quad \text{with} \quad A_0 = 6.18470415 \times 10^{-1}, \quad A_1 = 2.49235869 \times 10^{-2}, \quad A_2 = 2.33758602 \times 10^{-4}, \quad A_3 = -9.37151796 \times 10^{-6}. \tag{1}$$

The viscosity of ^{3}He can be written as

$$\eta = \frac{1}{5} n p_F \lambda = \frac{1}{5} n m \left(\frac{m*}{m}\right) v_F^2 \tau,$$
$$\text{thus} \tag{2}$$
$$\tau(P)T^2 = 5\eta(P)T^2 \frac{V_m}{5.009 \times 10^{-24} \times N_A} \frac{1}{(m^*/m)v_F^2},$$

with p_F and v_F the Fermi momentum and velocity respectively, n, the particle density, λ the quasiparticle mean free path, τ the viscous scattering time, V_m the molar volume, N_A, Avogadro's number, and m*/m the effective mass ratio. The mass of each ^{3}He atom, m = 5.009 $\times 10^{-24}$ g, is also needed to obtain the mass density.

The molar volume (in cm^3, with P in bar) is reproduced from Reference [5].

$$V_\mathrm{m} = \sum_{i=0}^{5} B_i P^i,$$
$$\text{with}$$
$$B_0 = 36.837231, \qquad B_1 = -1.1803474, \tag{3}$$
$$B_2 = 8.3421417 \times 10^{-2}, \quad B_3 = -3.8859562 \times 10^{-3},$$
$$B_4 = 9.475978 \times 10^{-5}, \quad B_5 = -9.1253577 \times 10^{-7}.$$

The effective mass was obtained by fitting a polynomial to the data in Reference [5].

$$\frac{m^*}{m} = \sum_{i=0}^{4} C_i P^i,$$

with

$$C_0 = 2.80, \qquad C_1 = 0.1292,$$
$$C_2 = -3.188 \times 10^{-3}, \quad C_3 = 9.372 \times 10^{-5},$$
$$C_4 = -1.03 \times 10^{-6}. \tag{4}$$

The Fermi velocity was obtained by fitting a polynomial to the data in Reference [5]

$$v_F = \sum_{i=0}^{4} D_i P^i,$$

with

$$D_0 = 59.8353052512899, \qquad D_1 = -1.99579000023344,$$
$$D_2 = 8.19564928730876 \times 10^{-2}, \quad D_3 = -2.10299440496278 \times 10^{-3},$$
$$D_4 = 2.13615981987922 \times 10^{-5}. \tag{5}$$

A polynomial fit to $T_c(P)$ on the PLTS scale is provided in Reference [4] and listed here

$$
\begin{aligned}
& T_{\mathrm{c,PLTS}} = \sum_{i=0}^{5} E_i P^i, \\
& \text{with} \\
& E_0 = 0.90972399274531, \qquad E_1 = 0.14037182852625, \\
& E_2 = -0.0074017331747577, \qquad E_3 = 2.8617547367067 \times 10^{-4}, \\
& E_4 = -6.5064429600510 \times 10^{-6}, \quad E_5 = 6.0754459040296 \times 10^{-8}.
\end{aligned} \tag{6}
$$

We use Supplementary Equations 1, 2, 3, 4, 5 to calculate $\tau_\eta T^2$ and then Supplementary Equation 2, 6 to calculate the quasiparticle scattering time at T_c, τ_c. We also need T_F to calculate the quantity $C(P)$ in Equation 4 of the main paper. We use Equation 6 in Reference [6] together with Supplemental Equation 4 to calculate T_F (K), using V_m in cc/mole.

$$
T_F = \frac{\hbar^3}{2mk_B(m^*/m)} \left(\frac{3\pi^2}{V_m}\right)^{2/3} = \frac{54.91}{m^*/m} V_m^{-2/3}. \tag{7}
$$

Values for the coefficient $\tau_\eta T^2$, τ_c, T_c, V_m, v_F, T_F and m^*/m at various pressures are listed in Supplementary Table 1. These values can be used along with the best fit determination of α = 0.434 and Γ = 40.8 to obtain values for C(P) in Equation 3 of the main paper, which also relates to $\delta Q_c/Q_c$, the maximum contribution to the excess Q at the transition temperature plotted in Figure 3 a) of the main paper.

The hydrodynamic regime ($\omega\tau \leq 1$), is distinct from the collisionless regime ($\omega\tau \geq 1$). In the

hydrodynamic regime, collisions are frequent compared to the frequency of the external excitation (*e.g.* pressure oscillation or shear frequency). In the collisionless regime, the excitation frequency exceeds the inverse mean time between collisions. In our experiment, the largest value of $\omega\tau_c$ is attained for P = 0 and T_c and is calculated to be $\approx$ 0.243 (see Supplementary Table 1). In the first observation of collisionless sound[7], the attenuation of first sound is $\approx 4\%$ below the expected normal state value for $\omega\tau \approx 0.25$. Thus, it is possible that at the lowest pressure, a portion of the deviation from $Q \propto T$ is due to a deviation from hydrodynamic behavior. However, such a contribution would be distributed over a range of temperature and not confined to the region near T_c. Additionally, the increase in T_c with pressure and the decrease of the coefficient ηT^2 (and τT^2) with pressure assures us that the departure from Fermi-liquid behavior cannot be accounted for by non-equilibrium effects due to any departure from the ideal $\omega\tau \ll 1$ regime.

The mean free path (λ_η) should be smaller than the viscous penetration depth δ=$(2\eta/\rho\omega)$, which is the distance over which the transverse velocity field in a fluid of density ρ, viscosity η, in contact with an object oscillating at a frequency ω, decays exponentially. The mean free path should also be smaller than the confinement size. These conditions are met well at high pressure, and marginally at low pressure (see Supplementary Table 1). Once again, the observation of the departure from Fermi-liquid behavior is strongest at high pressure, where the conditions for hydrodynamic behavior are well fulfilled.

Pressure [bar]	0	3	6	9	12	15	18	21	24	27	30
$\tau_\eta T^2$ [$\mu s m K^2$]	0.985	0.861	0.776	0.712	0.660	0.616	0.597	0.550	0.529	0.515	0.508
τ_c [μs]	1.19	0.533	0.328	0.234	0.182	0.149	0.127	0.111	0.100	0.0933	0.0887
T_c [mK]	0.9097	1.271	1.539	1.743	1.903	2.033	2.139	2.226	2.296	2.351	2.392
T_F [K]	1.77	1.66	1.56	1.49	1.42	1.36	1.31	1.26	1.22	1.17	1.13
V_m [cm^3]	36.84	33.95	32.03	30.71	29.71	28.89	28.18	27.55	27.01	26.56	26.17
m^*/m	2.80	3.16	3.48	3.77	4.03	4.28	4.53	4.77	5.02	5.26	5.50
v_F [m/s]	59.83	54.53	50.38	47.11	44.49	42.32	40.44	38.74	37.15	35.65	34.24
$\delta Q/Q_c$	0.0176	0.0244	0.0302	0.0343	0.0382	0.0416	0.0445	0.0474	0.0500	0.0544	0.0582
$\omega\tau_c$	0.243	0.109	0.0670	0.0478	0.0372	0.0304	0.0259	0.0227	0.0204	0.0191	0.0181
$\lambda_\eta(T_c)$ [μm]	71.2	29.1	16.5	11.0	8.10	6.31	5.14	4.30	3.72	3.33	3.04
$\lambda_\eta(3\,mK)$ [μm]	6.55	5.22	4.35	3.72	3.26	2.90	2.61	2.37	2.18	2.04	1.93
$\eta(T_c)$ [poise]	1.97	0.890	0.544	0.384	0.296	0.239	0.202	0.174	0.155	0.141	0.132
δ_c [μm]	154	99.2	75.3	61.9	53.5	47.4	43.0	39.6	36.9	35.0	33.5

Table 1: **Supplementary Table 1: Fermi liquid parameters**

Lists various quantities to estimate the fluctuation contribution in Equation 3, Main article, and for the discussion concerning mean free paths.

Supplementary Note 2: Background correction procedure

Outline of procedure

In this Supplementary Note, we describe details of the procedure we followed to correctly subtract the non-resonant background signal from our quartz tuning fork. The quartz fork is driven and detected through its in-built piezo electric capability. The distinguishing feature of our data on Q is the fact that we are able to track the quartz fork's Q continuously at all pressures. This is enabled by our use of a digital phase locked loop (PLL) that keeps the quartz fork on or near resonance. There is significant electrical coupling of the drive signal to the output side, with an attendant frequency dependent phase shift. The loop requires that the received vector signal $X(T), Y(T)$ from the lock in amplifier, has the non-resonant signal (feed through) subtracted from the received signal (See Supplementary Figure 3). When operated in vacuum, the fork's Q is high enough so that the received signal displays a Lorentzian response without background subtraction. When operated in liquid ^{3}He at low temperatures, the fork's Q can be as low as $\approx$10 leading to a broad response with a correspondingly small resonant signal requiring subtraction of the background signal for further analysis.

The subtraction procedure was carried out while gathering the data within the LabView Virtual Instrument environment. However, after the data was accumulated at several pressures, it became apparent that the original background calibration was insufficiently precise and that a post processing procedure would have to be followed. If the fitted background was used "as is", the result would be a non-Lorentzian resonance seen in the red trace in Supplementary Figure 5. (The

background subtraction and effects are shown in Supplementary Figures 3, 4, 5). An important condition imposed was that the Q *vs* T plot have a $Q(T=0)=0$ conforming with Fermi liquid behavior expectations. During our temperature sweep we adjusted the drive frequency when it differed from the inferred resonant frequency by 5 Hz. If the background subtraction is not performed correctly one finds small jumps in the inferred Q and resonant frequency. We used the elimination of these jumps as an additional constraint.

This Supplementary note details the procedure for all post-acquisition adjustments at one pressure (29.3 bar). Briefly, the procedure consisted of

1. A linear fit to the "as collected" Q *vs* T for temperatures between $1.2T_c$ and $1.4T_c$ yielded the intercept, $Q(0)_1$, listed as the first iteration of the correction procedure in Supplementary Table 2. $Q(0)_1$ was converted to signal units (Volts) using a constant k (defined later), and was subtracted from the $X(T)$ data, shown in Supplementary Figure 6. This "enforced" expectations of Fermi liquid behavior in the normal liquid ($\eta \to \infty$ as $T \to 0$).

2. The Nyquist trace $Y(T)$ *vs* $X(T)$ was plotted for each constant pressure cooldown. Circular arcs of constant Q were drawn for each drive-frequency reset, shown in Supplementary Figure 7a). The $X(T)$ data segments for a given fixed drive frequency were shifted to the nearest constant Q arc, shown in Supplementary Figure 7b). This was done to eliminate the slightly jagged character of the Q *vs* T plot. The mean displacement between $X(T)$ and its nearest constant Q arc (denoted as $\overline{\Delta Q_1}$) is the mean jump in Q found in the "as collected" data and is tabulated in Supplementary Table 2. $Q(T)$ was recalculated with the corrected

$X(T)$ data and the "as collected" $Y(T)$ data. This completes the first iteration of X offset corrections.

3. A linear fit to the recalculated $Q(T)$ data between $1.2T_c$ and $2T_c$ (or the highest temperature in the run), yielded $Q(0)_2$, the intercept in the second iteration of the X offset correction procedure (tabulated in Supplementary Table 2). $Q(0)_2$ was converted to Volts with the k constant, and was subtracted from the $X(T)$ data. The Nyquist trace was plotted, and circular arcs of constant Q were calculated for each frequency reset point. $X(T)$ data segments at fixed drive frequency were shifted onto the nearest constant Q arc. The mean displacement between $X(T)$ and its nearest constant Q arc, $\overline{\Delta Q_2}$, the mean jump in Q found in the second iteration of X offset corrections, is tabulated in Supplementary Table 2. The $Q(T)$ is recalculated with the corrected $X(T)$ data and the "as collected" $Y(T)$ data. This completes the second iteration of X offset corrections.

4. A linear fit to the doubly recalculated $Q(T)$ data for temperatures between $1.2T_c$ and $2T_c$ (or the highest temperature in the run), yields $Q(0)_3$, the intercept in the second iteration of the X offset correction procedure tabulated in Supplementary Table 2. $Q(0)_3$ was converted to Volts with the k constant, and was subtracted from the $X(T)$ data. The Nyquist trace was plotted, and the circular constant Q arcs were calculated and drawn at each frequency reset point. $X(T)$ data segments for a fixed drive frequency were shifted onto the nearest constant Q arc. The mean displacement between $X(T)$ and its nearest constant Q arc, $\overline{\Delta Q_3}$, (the mean jump in Q found in the third iteration of X offset corrections) is tabulated in Supplementary Table 2. The $Q(T)$ was recalculated with the corrected $X(T)$ data and the

"as collected" $Y(T)$ data. This completes the third iteration of X offset corrections.

5. The resonant frequency, $f_o(T)$, was recalculated after the three iterations of X offset corrections. It displayed discontinuous line segments, with jumps in $f_o(T)$ at each frequency reset point. (See Supplementary Figure 8). The sum of the jumps at each f_o was minimized by multiplying the "originally set" k in LabView by a multiplicative constant, k_{adj}, listed Supplementary Table 2. The final $Q(T)$ was recalculated with the new scaling constant $k_{adj} \times k$. A linear fit to the $Q(T)$ for temperatures between $1.2T_c$ and $2T_c$ (or the highest temperature in the run) was obtained, and its slope T_c is the Q_c reported in the main body of the paper. The intercept of this line is the final intercept quoted in Supplementary Table 2. Supplementary Figure 9 compares the final recalculation of $Q(T)$ and its linear fit, with the "as collected" $Q(T)$ and the linear fit calculated in step 1.

In the following, we provide more details accompanied by figures to clarify the procedure. Importantly, the fluctuation precursor is seen in the "raw data" before the various iterations at all pressures. The post data-acquisition procedure is needed to provide the "Fermi liquid background" behavior to scale the fluctuation contribution. We list the procedure and details so that other users may adapt it for their own investigations. Elimination or significant reduction of the non-resonant background signal is essential to resolve any finer detailed variation of the fluctuation contribution. Ultimately, the ability to continuously track the Q together with the high resolution thermometry enabled the fluctuation contribution to the viscosity of ^{3}He to be resolved in this experiment.

Background subtraction and first iteration.

As stated in the summary, we recorded values of $X(T), Y(T)$ obtained while driving a quartz resonator at a frequency, f_D near the fork's resonant frequency, f_0 immersed in ^{3}He. To calculate the Q and f_0, the non-resonant signal has to be first subtracted from the received signal.

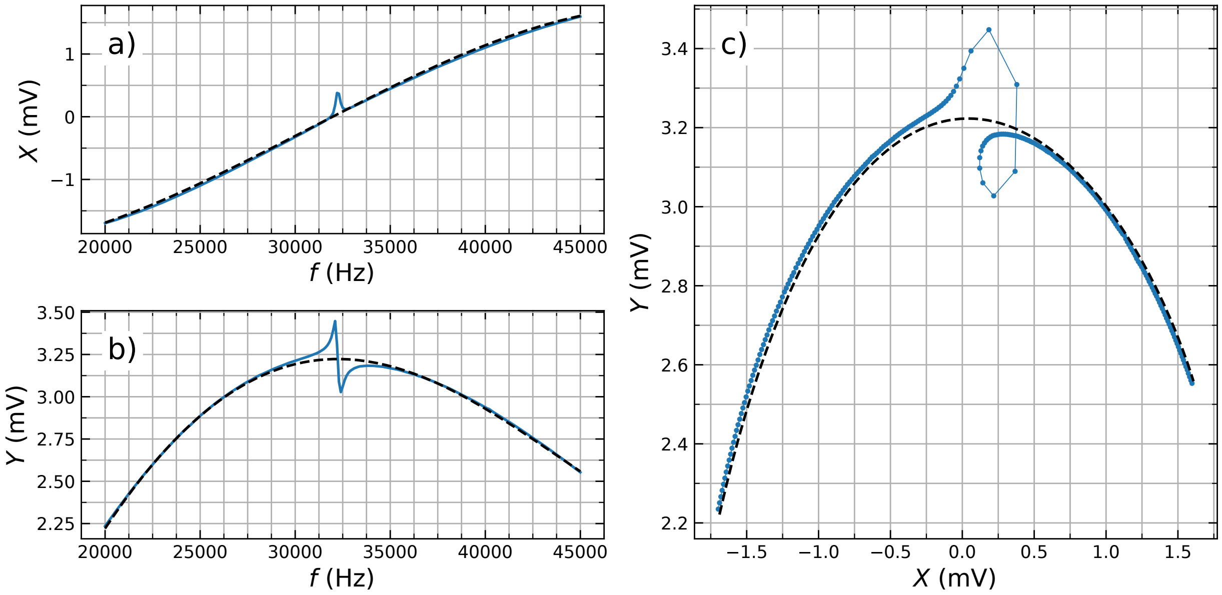

Supplementary Figure 3: **Quartz tuning fork background sweep.** The response of a quartz tuning fork at 8.1 mK and 29.3 bar over a wide frequency range. The dashed lines are the fitted 3rd order and 4th order polynomials for the X a) and Y b) responses after the correction noted to fit a Lorentzian to the fork's resonance (see Supplemental Figure 4). In panel c) we compare the fit (dashed line) to the observed complex response.

To effect this subtraction, we first carried out a sweep from 20 kHz through 45 kHz at $\approx$ 100 mK where the resonant line is narrow. This was done to assess the background and allow the fork

to be operated while cooling down to dilution refrigerator temperatures. At lower temperatures (8.1 mK shown in Supplementary Figure 3), we repeated this sweep. We then swept the frequency through the resonance over a frequency range spanning a few linewidths. We found that the X channel could be fit to a 3rd order polynomial, and the Y channel required a 4th order polynomial to adequately fit the background. After subtraction of these backgrounds, we plot the narrow range signal and obtain a Lorentzian fit for the resonance. A Nyquist plot with the real axis aligned along the X channel and the out-of-phase response aligned along Y reveal a near perfect circle plot. These X and Y signals (after subtraction of the fitted background), together with the associated Nyquist plot are shown in Supplementary Figure 4. In order to obtain a satisfactory Lorentzian fit a small ($\leq$ +0.05 mV) shift to the fitted background was needed. This increase in X accounts for the difference between the fit used (shown as a dashed line) and the data shown in Supplementary Figure 3. The Lorentzian is used to obtain the $Q = Q_R$, and the amplitude of the signal at resonance (A_R) is also noted. The previously mentioned constant k is defined as $k = Q_R/A_R$. (The value of k used in the LabView VI was likely not accurate enough and necessitated adjustments described in the following sections). Together with the $X(T)$ and $Y(T)$ (X, Y values after subtraction of the background at any temperature T), these constitute the inputs to the determination of the resonant frequency $f(T)$ and the $Q(T)$ using the equations[8],

$$Q(T) = \frac{X(T)^2 + Y(T)^2}{X(T)} \left(\frac{Q_R}{A_R} \right) \tag{8}$$

$$f(T) = f_D(T)\left(1 + \frac{Y(T)}{X(T)}\frac{1}{2Q_T}\right) \tag{9}$$

where $f_D(T)$ is the drive frequency at any temperature, T. These equations form the basis of the PLL that maintains the fork within $\pm$ 5 Hz of resonance and were used to calculate the "raw" values of $Q(T)$ and $f(T)$.

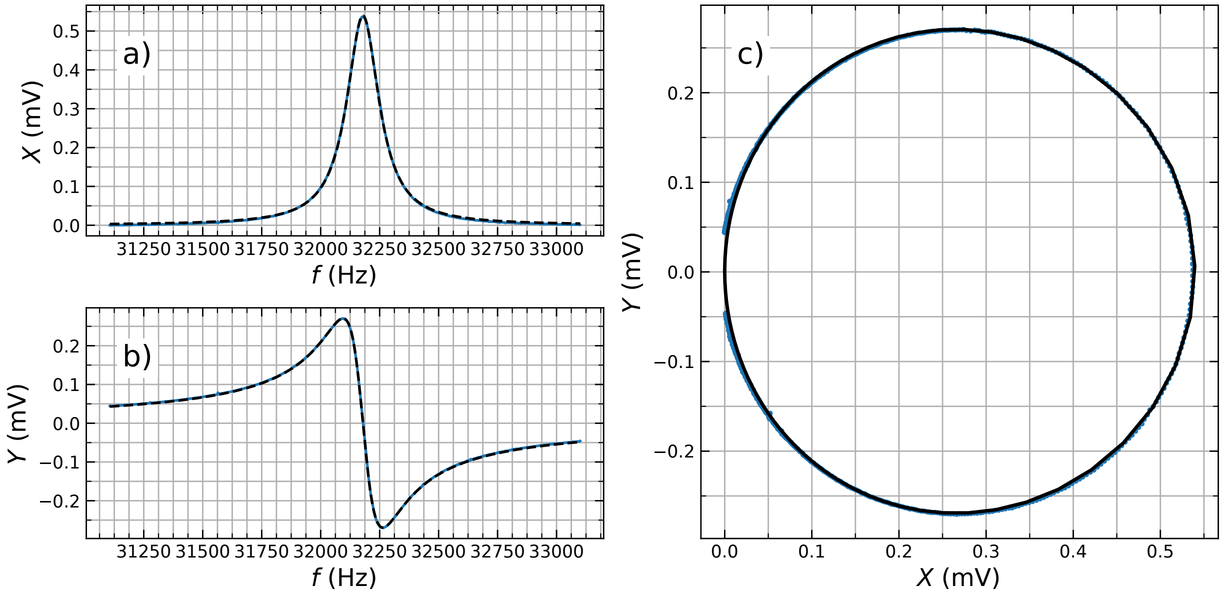

Supplementary Figure 4: **Quartz tuning fork Lorentzian response.** The response of a quartz tuning fork at 8.1 mK and 29.3 bar after subtraction of the background. The black (dashed/solid) lines on the (left/right) are the fitted curves to a Lorentzian response.

When we apply the same background subtraction to a frequency sweep at 4.9 mK (obtained a few days later), where the Q is further reduced, the Nyquist response within the linewidth is horizontally off-center. This offset in the X background is not systematically temperature dependent.

Instead it appears that there is a small frequency dependence to the background (corresponding to a first order term) that is not captured in our background subtraction procedure.

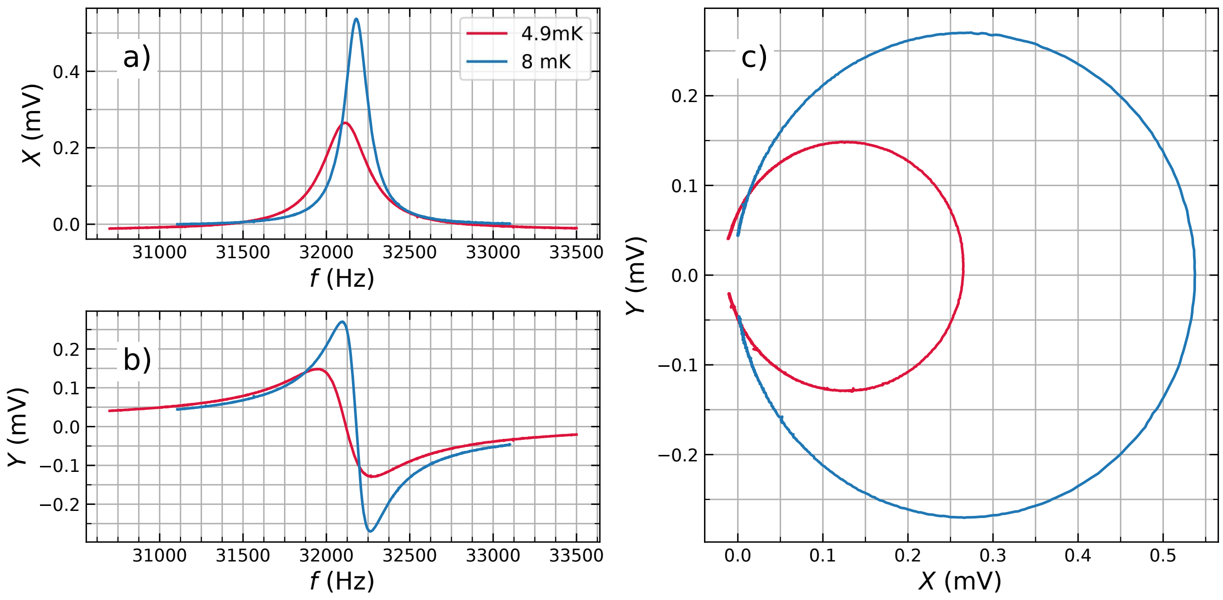

Supplementary Figure 5: **Quartz tuning fork Lorentzian responses at 8.1 mK and 4.9 mK.** The response of a quartz tuning fork at 29.3 bar taken at 8.1 mK (blue) compared to the response at 4.9 mK (red), both obtained after subtraction of the fitted background signal obtained at 8.1 mK (See Supplementary Figure 4c)). The Nyquist plot for the 4.9 mK signal shows an offset from the origin. This is also visible as a negative response for the X signal at 4.9 mK (red) in a).

Because the origin of this shift in background was not evident when the data was being acquired, we introduced a workaround to compensate for this shift. We collect data in a limited period of time (typically 1 day) while ensuring minimal thermal gradient between the thermometer immersed in the ^{3}He and the quartz fork. In practice, this limits us to temperature sweeps within $\approx$ 40% of T_c. Following this procedure, we observe an artificial intercept ($Q \neq 0$ at T = 0) in

the temperature dependence of the Q in ^{3}He (Supplementary Figure 6). After subtraction of this intercept ($Q(0)_1$), the inferred Q is plotted as the green line in Supplementary Figure 6. Intercepts of this magnitude or smaller were observed for all the different pressure runs. A list of the offset $Q(0)_1$ values subtracted for each pressure is shown in Supplementary Table 2.

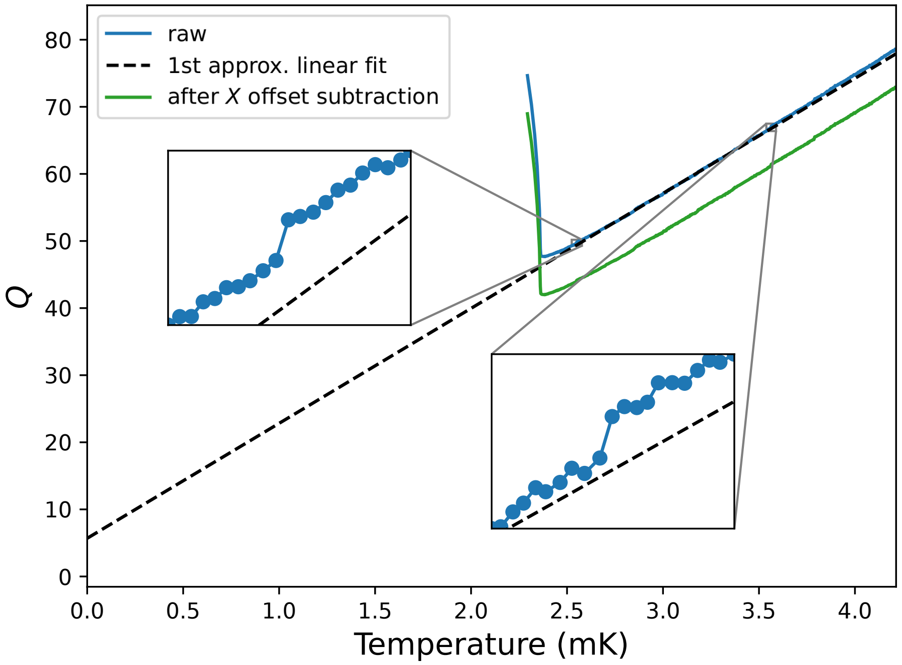

Supplementary Figure 6: Q **vs T before and after offsets at** $P = 29.3$ **bar.** Temperature dependence of Q (blue line) inferred following subtraction of the non-resonant signal background. The dashed line is a linear fit on a 10 percent random sample between $1.2T_c$ and $1.4T_c$. The temperature fitting range was kept narrow in order to avoid fitting jumps in the data. After subtraction of the offset, we plot the resulting Q vs T in green.

As the ^{3}He is cooled, due to the increased viscosity, the mass of ^{3}He coupled to the fork

changes, resulting in a decrease of the resonant frequency. Since the PLL operates at a fixed drive frequency, we reset the drive frequency once f_D -$f_0(T) \geq 5$ Hz. However, because of the offset in X that shifted the Q intercept, Equations 8, 9 are no longer exactly valid. This leads to artificial jumps in the inferred Q produced when the drive frequency is reset (see the insets to Supplementary Figure 6). We note that it was difficult to calculate a line of best fit for linear data broken up into slightly offset line segments as seen in Supplementary Figure 6. This was remedied after the Q continuity correction.

A line was fitted to the $Q(T)$ data, and the $Q(0)_1$ value was converted to a voltage, X_{offset}. The $Q(0)_1$ values vary between $-1 < Q < 6$ with pressure and are listed in Supplementary Table 2. After subtracting the X_{offset} from the raw fork response, we carry out the corrections to achieve the continuity of Q as described next.

In Supplementary Figure 7 (a), we plot the values of $X(T)$, $Y(T)$ obtained during a cooldown at 29.3 bar. The traces are broken up into segments of data collected at a fixed drive frequency. At each transition the drive frequency was reset by the PLL to be on resonance ($Y(T)$ = 0). In the top panel of Supplementary Figure 7, at each point where a frequency reset is triggered, a circular arc is traced. This arc of constant Q corresponds to a segment of a Nyquist plot for a circle of diameter corresponding to the inferred Q factor just before the reset, centered at (X, Y) corresponding to Q/2, 0. After the frequency reset, the green trace corresponding to the uncorrected data, systematically deviates away from the arc. This indicates that the background fit used has a small systematic frequency dependent error that was not resolved in the fitting procedure described earlier to obtain

the Lorentzian fit shown in Supplementary Figure 4. To correct for this offset, the next segment of the data is shifted to the preceding arc of constant Q. For the specific case of the 29.3 bar data shown here, each segment was shifted by a positive increment to X, corresponding to a positive shift in Q. We find that the individual changes to $\overline{\Delta Q_1}$ are of order 0.2 ± 0.1. The resulting corrected trace is shown in red in the bottom panel of Supplementary Figure 7. This concludes the first iterative correction.

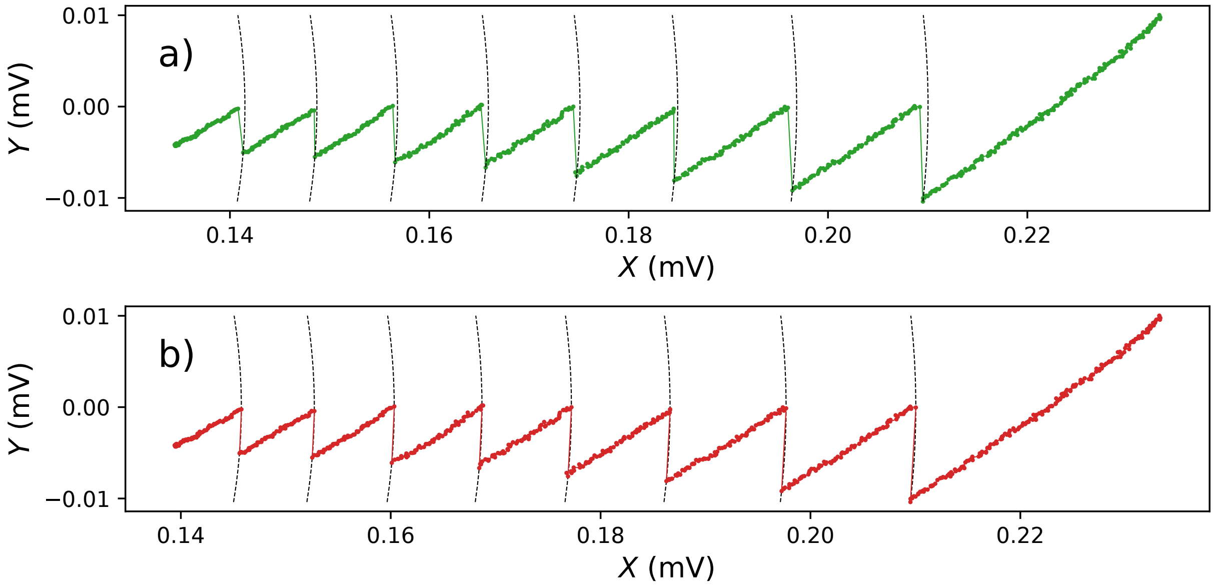

Supplementary Figure 7: **Nyquist trace during a cooldown at P = 29.3 bar.** The top trace shows the observed trace of $Y(T)$ vs $X(T)$ data taken while cooling before correction (green). As the temperature decreases, the $X(T)$ decreases, and $Y(T)$ also decreases corresponding to a decrease in the resonant frequency. Also shown are individual segments of circles (Nyquist plots) originating at X = 0, Y = 0 that pass through the last point in $X(T)$, $Y(T)$ before the frequency is reset. The lower panel depicts the trace (red) of $X(T)$ vs $Y(T)$ after adjustment described in the text.

Second and Third Iterative Correction.

Because of the cumulative change in the Q accompanying each reset of the drive frequency, the T = 0 intercept seen in Supplementary Figure 3 would be changed. Therefore, a further two iterations to obtain the offsets $Q(0)_2$ and $Q(0)_3$ and Q continuity corrections yielding $\overline{\Delta Q_2}, \overline{\Delta Q_3}$ were carried out to minimize the $T = 0$ intercept and any remaining discontinuities in Q across resets of drive frequency. These linear fits were extended to between 1.2 T_c and $2T_c$ (or the highest temperature that data at a given pressure was acquired at). The final corrected data and linear fit is shown as the color coded trace in Figure 1 of the main paper, and was used in all further analysis detailed in the main paper. The values for $Q(0)_2$, $Q(0)_3$ and $\overline{\Delta Q_2}, \overline{\Delta Q_3}$ are listed in Supplementary Table 2.

Correction to k.

The frequency dependent correction described in the previous sections is essentially confined to $X(T)$. Consequently, the raw inferred resonance frequency $f_0(T)$ is continuous with temperature (blue trace in Supplementary Figure 8). However, after making corrections to the X component of the response as detailed in this Supplementary Note, the inferred frequency is no longer continuous across changes in drive frequency (red trace in Supplementary Figure 8). A correction is needed to k (the difference in the value of the inferred frequency from the drive frequency depends on the value of k - See Supplementary Equations 8, 9). Note that $Y(T) \ll X(T)$, so that $Q(T) \approx X(T) \times k$. Therefore, we apply a multiplicative constant $k_{adj} = k'/k$, where k' is the new voltage to Q conversion factor. The k_{adj} is found by minimizing the sum of all the jumps at a frequency reset,

resulting in the dashed orange trace in Supplementary Figure 8 that aligns well the raw resonance frequency. In a few runs (8 bar, 5 bar and 2 bar) the drive frequency was held fixed throughout the temperature sweep. Consequently, there are no values for $\overline{\Delta Q_1}, \overline{\Delta Q_2}, \overline{\Delta Q_3}$ in the table. In these runs, the zero temperature offset was subtracted, and the k_{adj} was found by minimizing the difference in the original raw f_o and the f_o upon a X offset subtraction based on a zero temperature intercept.

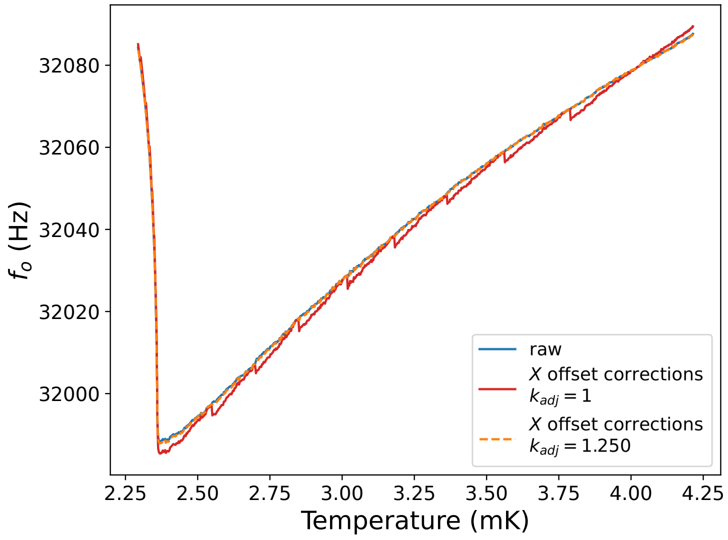

Supplementary Figure 8: **Resonant Frequency** *vs* **Temperature at** $P = 29.3$ **bar.** The blue trace shows the raw data for resonant frequency plotted against the temperature, obtained from the fit to Q at 8.1 mK. Following offsets of Q, the resulting plot (orange) displays discontinuities. This is resolved by appropriate changes to k_{adj} restoring $f_0(T)$ (dashed red line) to the original values.

After following these steps, we plot the corrected results for Q vs T and δQ vs T alongside the uncorrected raw data in Supplementary Figures 9, 10. We also show the full extent of all

temperatures and pressures measured with corrections applied in Supplementary Figure 11. Individual plots similar to Supplementary Figure 9 for 27 bar, 23 bar, 20 bar and 15 bar are shown in Supplementary Figure 12 and for 8 bar, 5 bar, 2 bar and 0.5 bar are shown in Supplementary Figure 13.

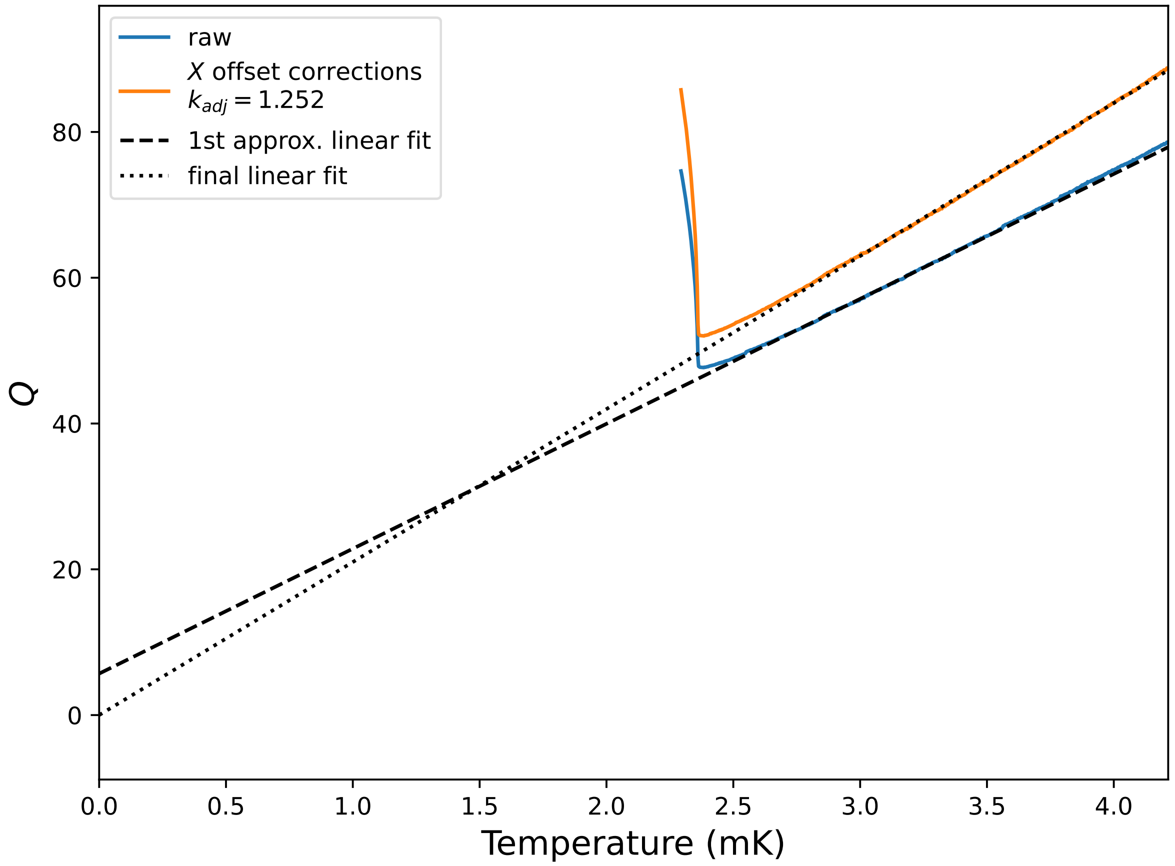

Supplementary Figure 9: Q *vs.* **Temperature before and after** X **offset corrections** $P = 29.3$ **bar**. We show the Q before (blue) and after (orange) the X offset and k correction were applied. The plot in shown in gold here appears in Fig 1 a) of the main paper. The dashed line is a linear fit on the raw data in the 1st iteration of the correction procedure. The dotted line is a linear fit on the fully corrected data.

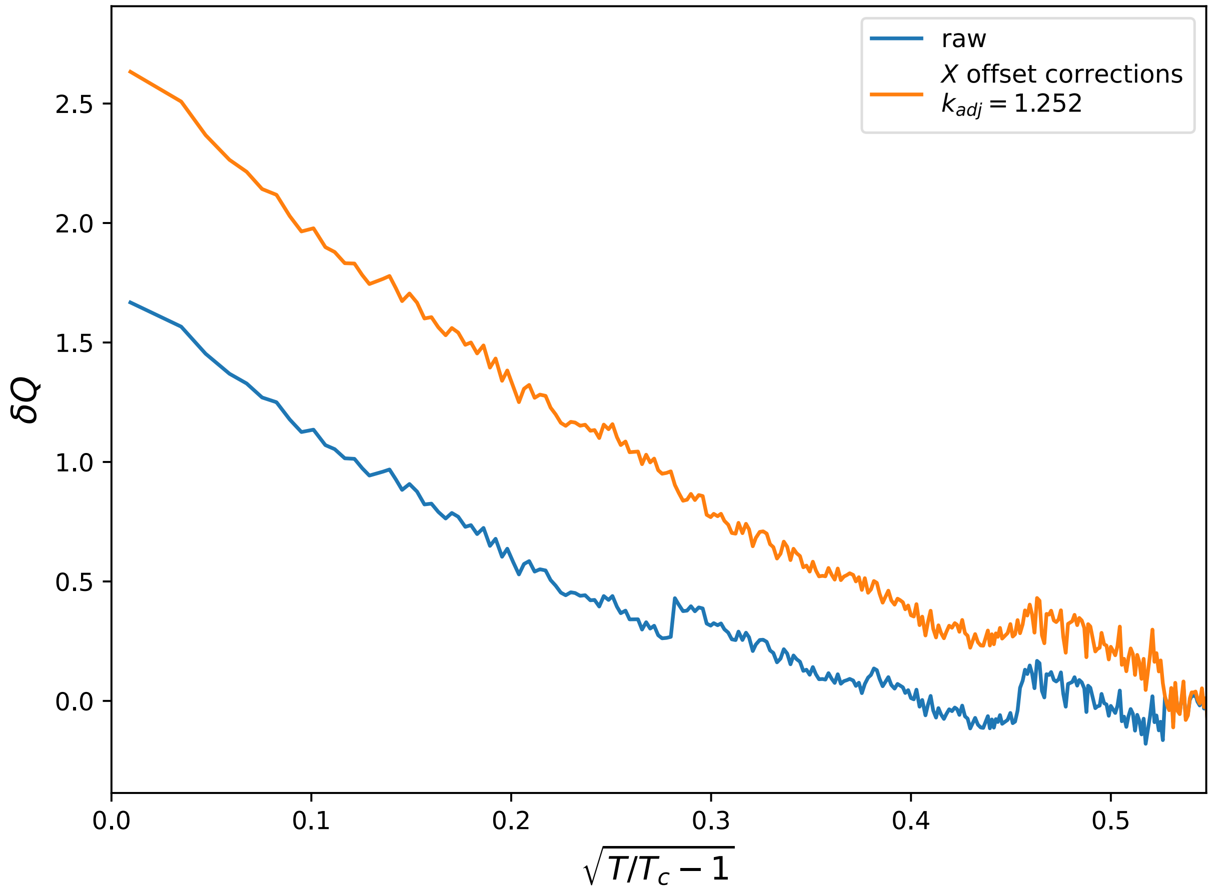

Supplementary Figure 10: δQ *vs.* **the square root of the reduced temperature before and after offsets at** $P = 29.3$ **bar.** We show the δQ before (blue) and after (gold) the correction offsets were applied. The plot in shown in gold here appears in Figure 2a) of the main paper.

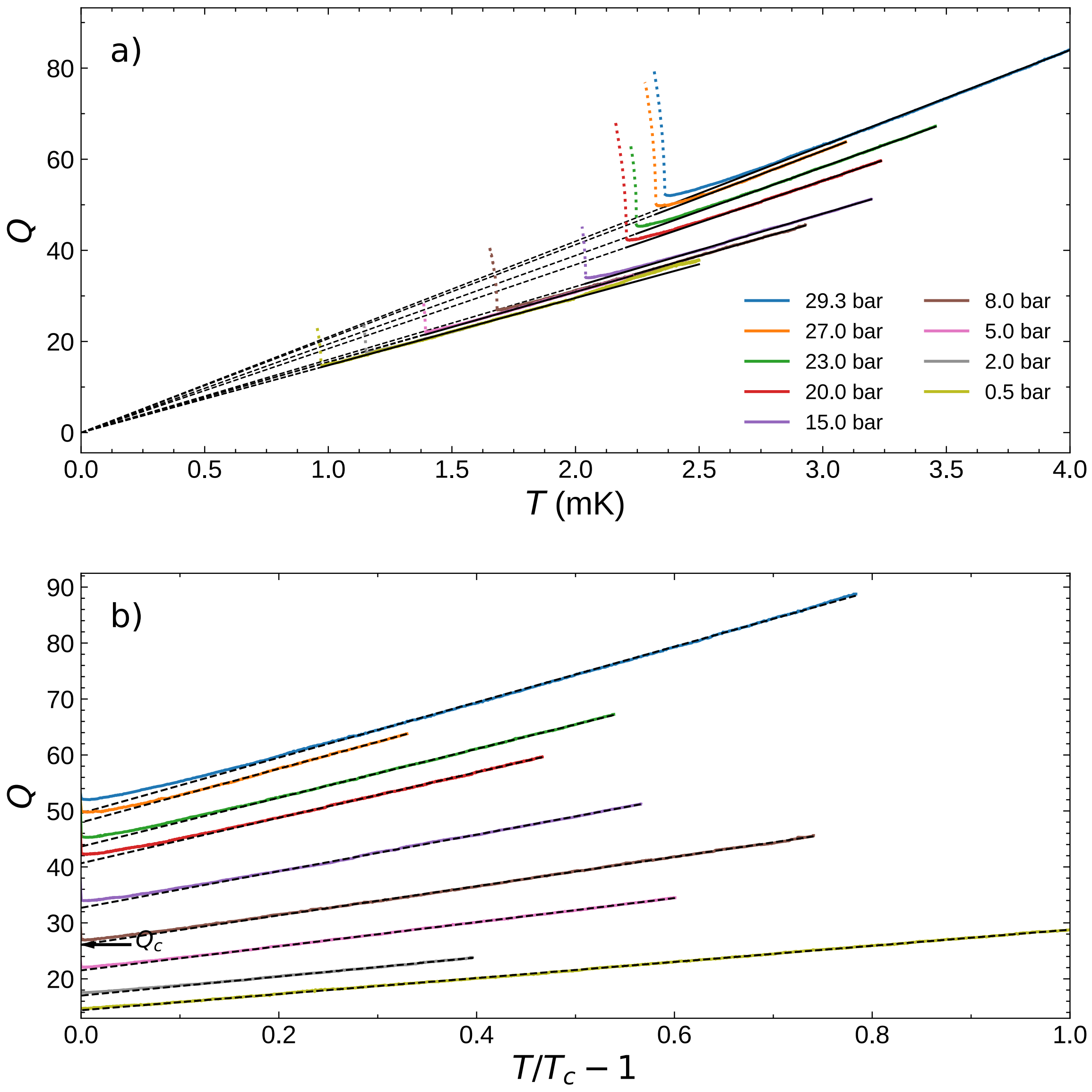

Supplementary Figure 11: **Corrected Q vs. temperature of all pressures over a larger temperature range.** We show the corrected Q vs. temperature data up to the highest temperature data was taken for each pressure. a) Corrected Q vs temperature, where the dashed lines are linear fits. The dotted colored line is the fork response below T_c. The dashed black lines are the linear fits extrapolated below T_c. b) Q versus a reduced temperature scale.

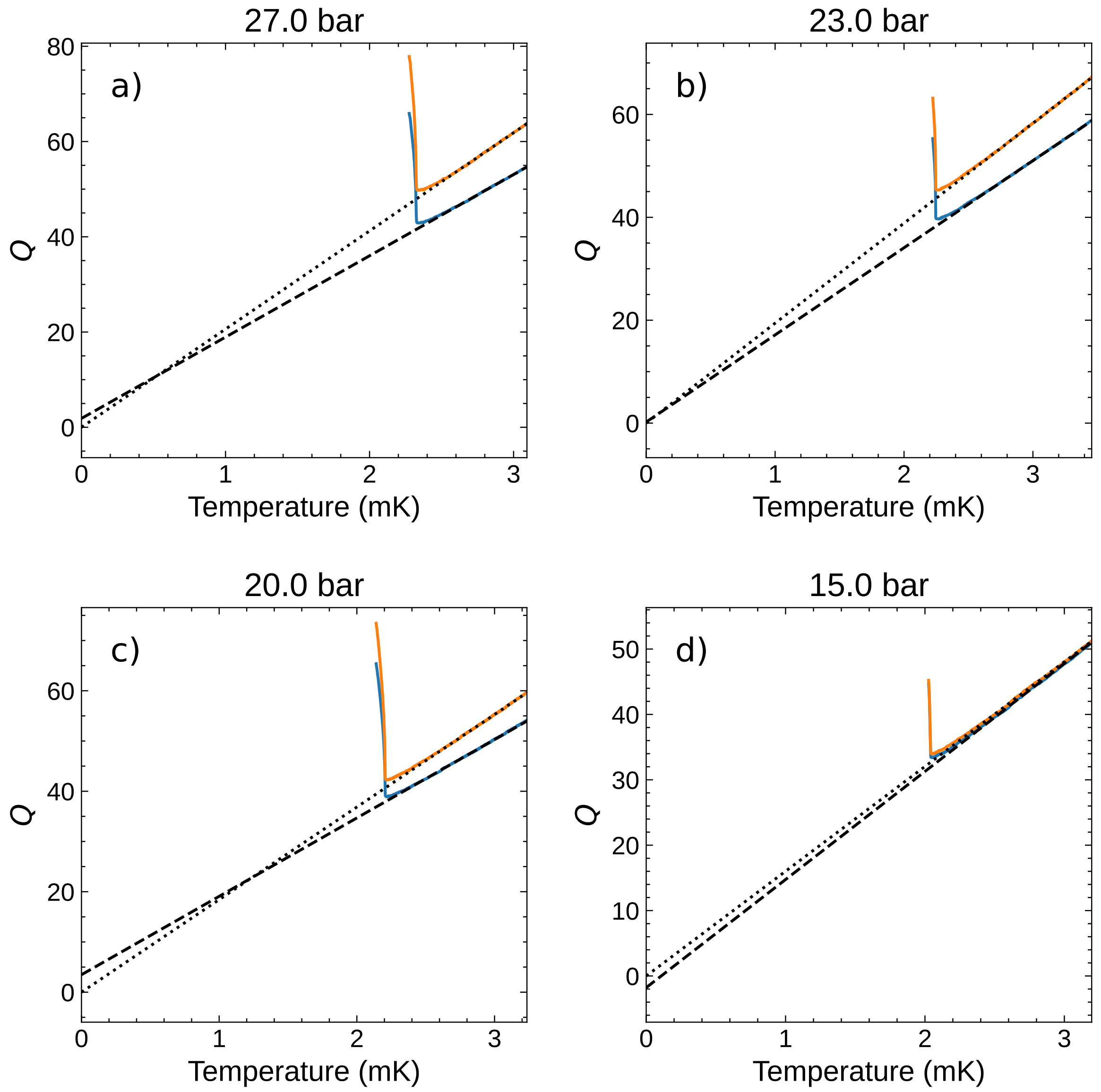

Supplementary Figure 12: **Corrected Q vs. temperature for a) 27 bar, b) 23 bar, c) 20 bar, and d) 15 bar.** The legend for the traces plotted in each section of the grid are equivalent to Supplementary Figure 9. The plot compares the corrected data (orange), to the "as collected" data (blue). The k_{adj} constants for each run can be found in Supplementary table 2. The dashed line is a linear fit to the uncorrected data, and the dotted line is the final linear fit to the corrected data.

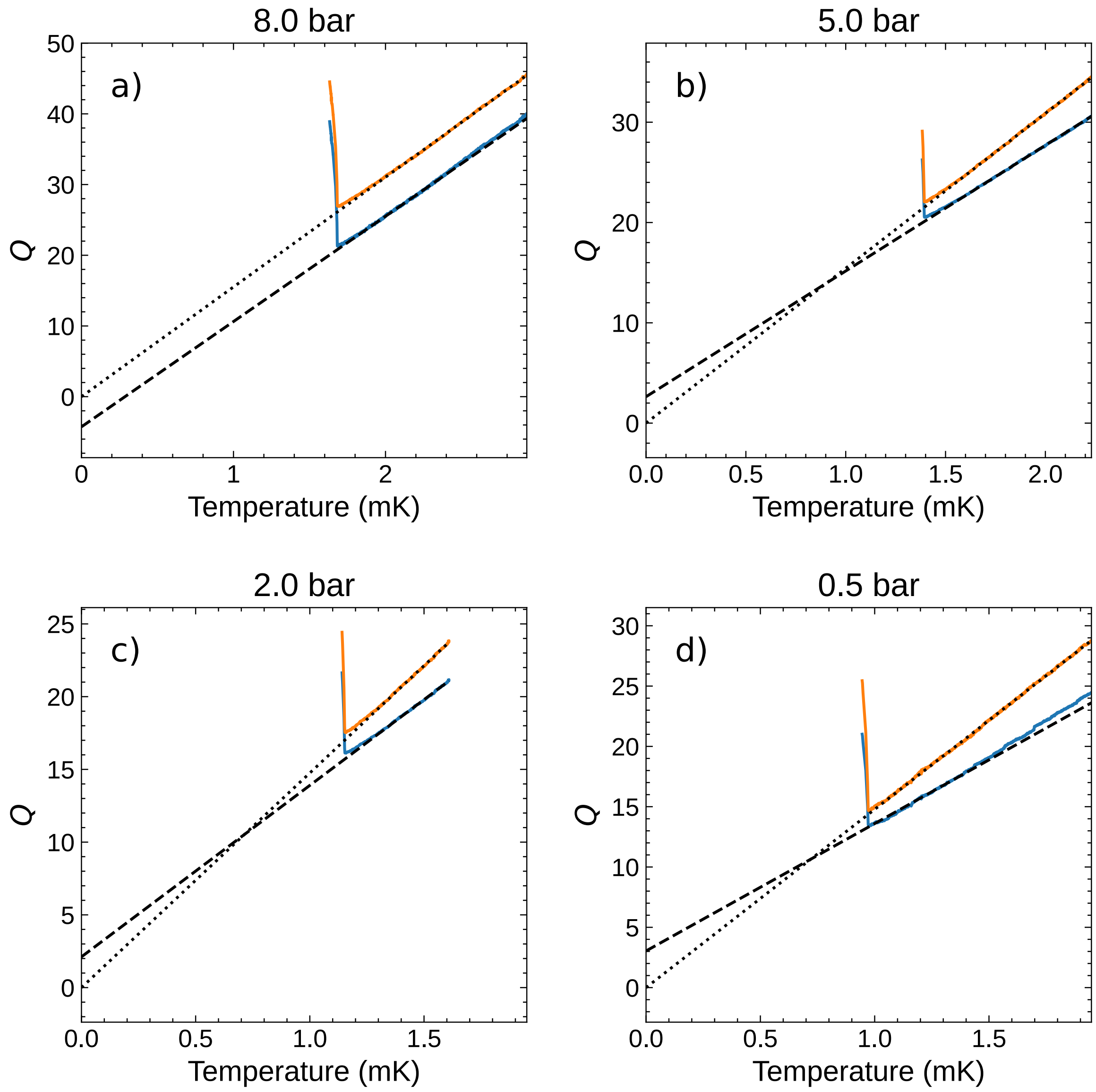

Supplementary Figure 13: **Corrected Q vs. temperature for a) 8 bar, b) 5 bar, c) 2 bar, and d) 0.5 bar.** The legend for the traces plotted in each section of the grid are equivalent to Supplementary Figure 9. The plot compares the corrected data (orange), to the "as collected" data (blue). The k_{adj} constants for each run can be found in Supplementary table 2. The dashed line is a linear fit to the uncorrected data, and the dotted line is the final linear fit to the corrected data.

P (bar)	final $Q(0)$	k_{adj}	$Q(0)_1$	$Q(0)_2$	$Q(0)_3$	1st $\overline{\Delta Q_1}$	$\overline{\Delta Q_2}$	$\overline{\Delta Q_3}$
29.3	0.00252	1.25	5.69	1.95	0.059	-0.231	-0.0025	-0.000103
27	4.13e-05	1.22	1.86	0.594	0.00431	-0.00353	-9.13e-05	-7.53e-07
23	-1.3e-05	1.14	0.208	-0.182	-0.00154	-0.00112	3.32e-05	2.23e-07
20	-3.81e-05	1.18	3.48	-0.0172	-0.00146	-0.0829	0.00019	1.35e-06
15	0.000169	1	-1.78	1.47	0.0157	-0.024	-0.000407	-4.22e-06
8	-1.03e-07	1	-4.26	-1.36	9.78e-06	0	0	0
5	0.000111	1.23	2.63	0.0485	-0.00209	0	0	0
2	-5.64e-05	1.25	2.12	-0.0543	0.00154	0	0	0
0.5	2.64e-05	1.42	3.04	0.0837	-0.00319	0.0696	-0.00018	9.17e-07

Table 2: **Supplementary Table 2: Background Subtraction.** The table lists the pressures, the final value of $Q(T = 0)$, the adjusted value of k (the multiplicative factor applied the conversion factor k from signal amplitude to Q), the $Q(T = 0)$ for three iterations of the correction procedure, and the the average jump in Q after a frequency reset for three iterations.

Background in the low-Q regime

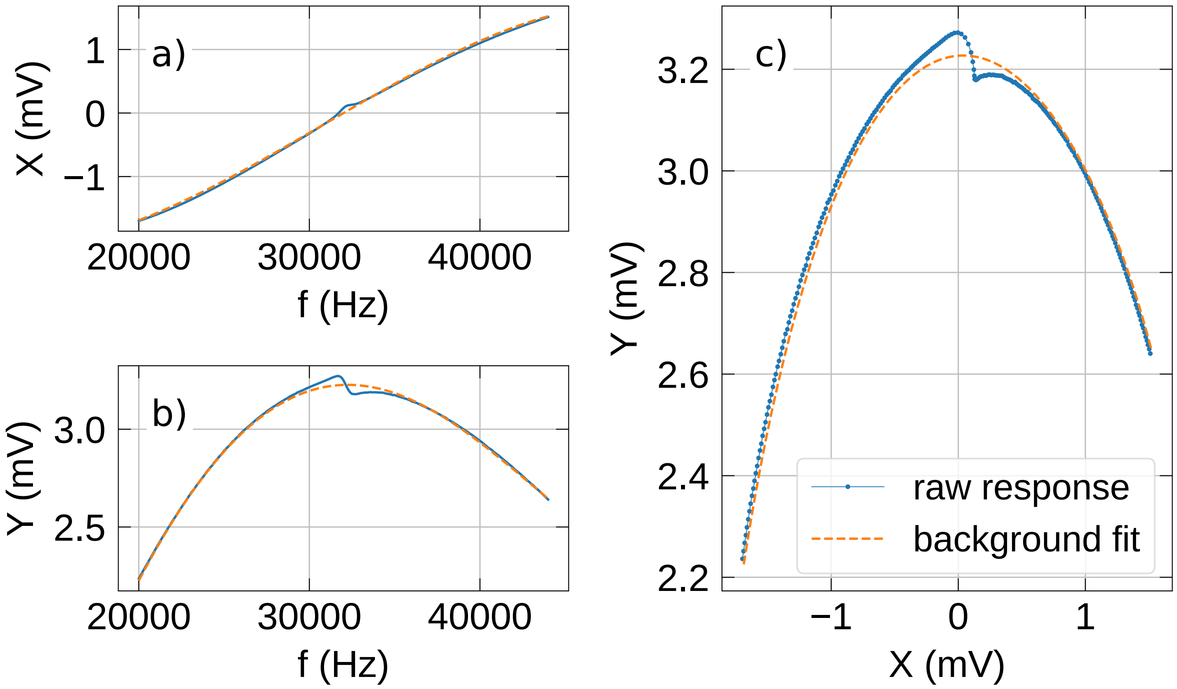

Supplementary Figure 14: **Background subtraction over a wide frequency range in the low Q regime.** $T = 2.5$ mK and $P = 0.56$ bar. a) The X response of the quartz fork fitted to a 3rd order polynomial. b) The Y response of the quartz fork fitted to a 4th order polynomial. c) The fork response over a wide frequency range in a Nyquist plot, together with the fit (orange dashed line).

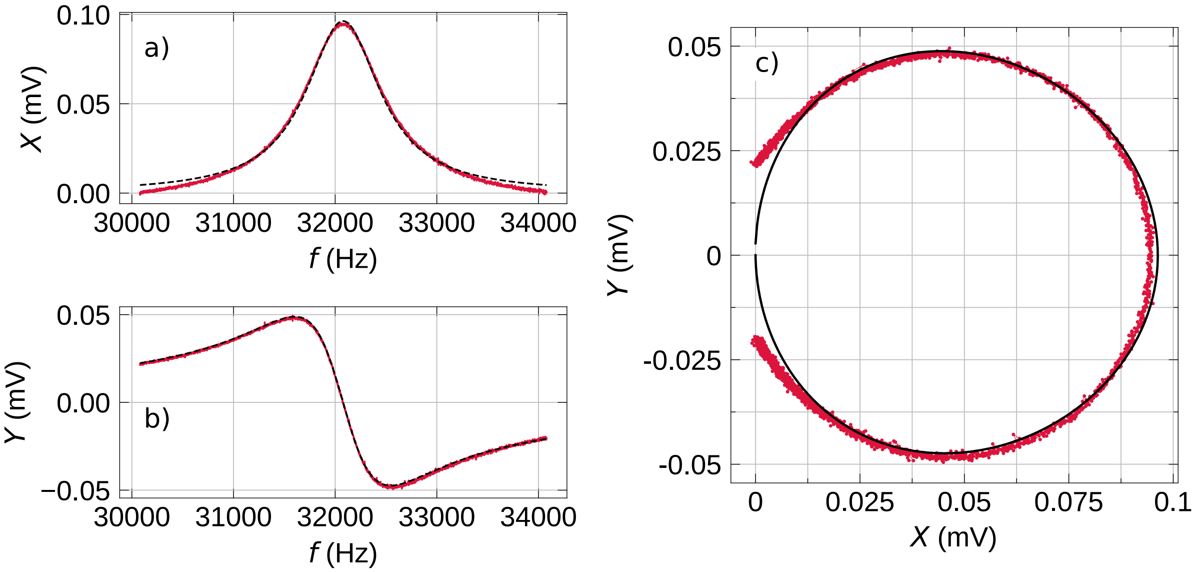

Supplementary Figure 15: **Background subtraction over a narrow frequency range in the low Q regime.** $T = 2.5$ mK and $P = 0.56$ bar. The Q-factor from this fit is ≈ 35, and the fitted resonance is $f_0 = 32079$ Hz (linewidth, $\Delta f = 916$ Hz). a) The X response of the quartz fork fitted to the real part of a complex Lorentzian. b) The Y response of the quartz fork fitted to the imaginary part of a complex Lorentzian. c) The quartz fork response and fit in a Nyquist plot.

In addition to the background presented in Supplementary Figures 3, 4, we carried out a frequency sweep to fit the background at low temperature and low pressure. Supplementary Figure 15a) shows poor agreement between the real response and the fit to a Lorentzian. The fit deviation in the X component is observed in the Nyquist plot in Supplementary Figure 15 a) and c) as well. The fork's response is small in the low-Q regime. The broader response due to the low Q and a temperature dependent background, is responsible for a poor fit. In Supplementary Figure 16 we

show the consequence of using a high temperature and high pressure fit to data obtained in the low Q regime.

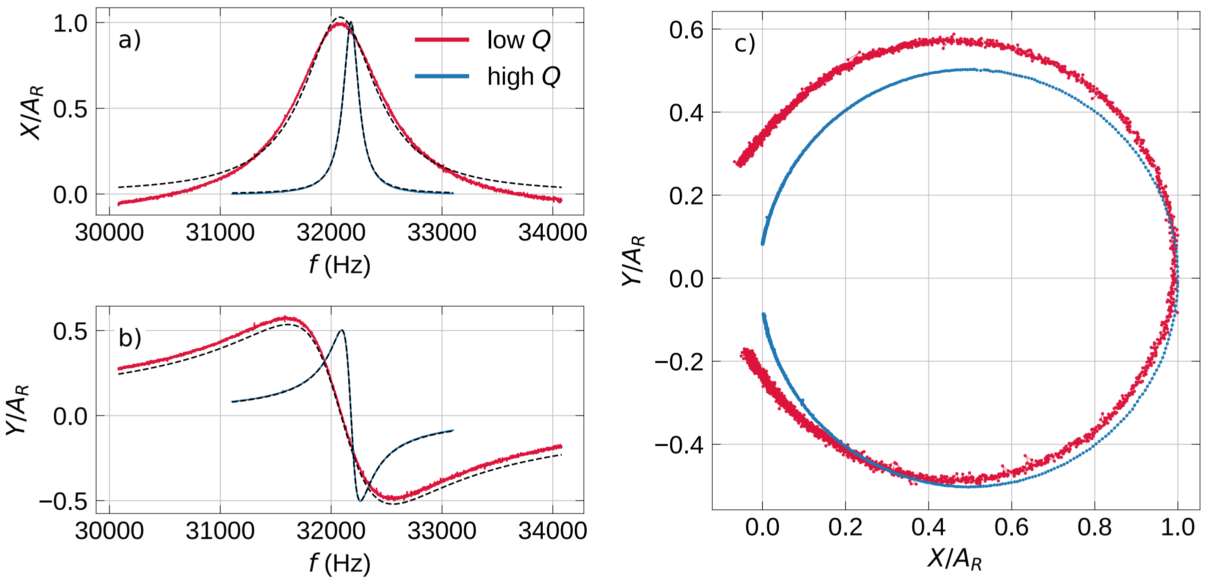

Supplementary Figure 16: **Low Q sweep with a high Q/high temperature and pressure background.** The blue trace is the response from Supplementary figure 4 minus the background obtained from the wide response in figure 3, at $T = 8.1$ mK and P = 29.3 bar. The red trace is the fork response at $T = 2.5$ mK, $P = 0.56$ bar, minus the same high temperature and high pressure background as the blue trace. a) The X responses of the quartz fork fitted to the real part of a complex Lorentzian, and normalized by the resonant amplitude. b) The Y responses of the quartz fork fitted to the imaginary part of a complex Lorentzian and normalized by the resonant amplitude. c) The quartz fork responses in a Nyquist plot normalized by the resonant amplitude.

1. Parpia, J. M., Sandiford, D. J., Berthold, J. E. & Reppy, J. D. Viscosity of normal and superfluid helium three. *J. Phys. Colloques C6* **39**, C6–35–C6–36 (1978). URL `https://doi.org/10.1051/jphyscol:1978617`.

2. Parpia, J. *The Viscosity of Normal and Superfluid* ^{3}He. Ph.D. thesis, Cornell University (1979).

3. Rusby, R. *et al.* Realization of the ^{3}He Melting Pressure Scale, PLTS-2000. *J. Low Temp. Phys.* **149**, 156–175 (2007). URL `https://doi.org/10.1007/s10909-007-9502-y`.

4. Tian, Y., Smith, E. N. & Parpia, J. Conversion between ^{3}He melting curve scales below 100 mk. *Journal of Low Temperature Physics* **184**, 1573–7357 (2022). URL `https://doi.org/10.1007/s10909-022-02721-z`.

5. Greywall, D. ^{3}He specific heat and thermometry at millikelvin temperatures. *Phys. Rev. B* **33**, 7520–7538 (1986). URL `http://dx.doi.org/10.1103/PhysRevB.33.7520`.

6. Greywall, D. S. Specific heat of normal liquid ^{3}He. *Phys. Rev. B* **27**, 2747–2766 (1983). URL `https://link.aps.org/doi/10.1103/PhysRevB.27.2747`.

7. Abel, W. R., Anderson, A. C. & Wheatley, J. C. Propagation of zero sound in liquid ^{3}He at low temperatures. *Phys. Rev. Lett.* **17**, 74–78 (1966). URL `https://link.aps.org/doi/10.1103/PhysRevLett.17.74`.

8. Morley, G. W. *et al.* Torsion pendulum for the study of thin ^{3}He films. *Journal of Low Temperature Physics* **126**, 557–562 (2002). URL `https://doi.org/10.1023/A:1013767117903`.
